# Effect of Placement Technology on the Bond Strength Between Two Layers of Self-Compacting Concrete

**DOI:** 10.3390/ma13153330

**Published:** 2020-07-27

**Authors:** Piotr Dybeł, Milena Kucharska

**Affiliations:** Department of Geomechanics, Civil Engineering and Geotechnics, AGH University of Science and Technology, 30-059 Cracow, Poland; kucharska@agh.edu.pl

**Keywords:** multilayer casting, self-compacting concrete, bond strength, lift line, placement technology

## Abstract

Self-compacting concrete (SCC) should generally be placed continuously, but it is not uncommon for contractors to be forced to use interruptions in concrete works due to delivery delays. The multilayer casting of SCC can cause weak bond conditions in the contact area of subsequent layers. Methods of preventing cold joint or lift line formation for normal concretes are not suitable for self-compacting concretes. This article provides research on the effect of multilayer casting technology on the bond strength between two layers of SCC. Three technological variants of connecting successive layers of SCC mixture on beam elements were analyzed: The free flow of the mixture, dropping the mixture from a greater height, and mechanical disturbance of the first layer. Three delay times were applied: 30, 45, and 60 min between two layers of SCC. In general, the research revealed that, regardless of the multilayer casting variant, the bond strength between two layers decreased as the delay time was extended. The best performance and the lowest drop in bond strength were obtained for samples with a mechanically disturbed first layer, independent of the delay time. This method gave similar results to a reference element made without a break in concreting. It was also demonstrated that current recommendations and standard guidelines for multilayer casting appear to be insufficient for ensuring an adequate bond between layers.

## 1. Introduction

One of the most valued features of concrete is the possibility to form a complete structure from it—known as a monolithic reinforced concrete structure. During the creation of monolithic structures, it is recommended and highly desirable to carry out works continuously. However, it is often not possible to perform all of the works at one time. Contractors are forced to take breaks in concrete mix placement due to delivery delays or the given technology of the construction. Interruptions in concrete works can result in the formation of cold joints or lift lines between successive layers. As a result, this can lead to a deterioration of the strength properties of the hardened concrete, its durability, and the surface finish. Interruptions in concrete works, especially when combined with rapid workability loss of the mixture, results in visible marks in the contact areas and discoloration of the concrete surface [1].

For normal concrete (NVC), compaction is carried out by vibrations between successive layers of concrete mix. According to the normative guidelines [2,3], each contact surface of the successive layers should be vibrated by placing a vibrator head approximately 10 cm deep into the previously placed and compacted layer. In the case of the self-compacting concrete (SCC) mixture, this technology cannot be implemented directly, as the crux of self-compacting concrete is to eliminate the need for mechanical compaction. Moreover, exposing the mixture to even weak vibrations disrupts its internal stability and leads to very strong segregation. For this reason, vibrating devices should not be used when placing SCC, as well as near the concrete placement site. Because of its thixotropic character, SCC can be placed onto previously placed SCC that has gelled, but not yet achieved an initial set. Yet, whenever possible, SCC should be cast continuously and in thick layers so that no fresh SCC is placed on concrete that has hardened; otherwise, the successive layers will not mix properly and, as vibrating is prohibited in the case of SCC, a weak interface between the concrete layers may appear in the final structure, called a lift line or fold line. This should not be confused with a cold joint that occurs when the successive layer is cast on top of an already set concrete surface, which is prevented by increasing the roughness of the primary layer.

Thixotropy is interpreted as an increase of viscosity while at rest and decrease of viscosity when the material is exposed to constant shearing stress [4]. It is reversible and associated with the so-called structural buildup at rest (SBR), which occurs when the given shear rate is removed. Both of these phenomena significantly affect a number of different properties of concrete, including the pumping, segregation resistance, surface quality, formwork pressure, and bond strength between successive layers in multilayer casting [5,6,7,8,9]. While increasing the thixotropy of the mixture and decreasing the concreting speed are recommended to reduce the high pressure of self-compacting mix on a formwork [10,11], they simultaneously contribute to increasing the structural buildup at rest. This phenomenon favors the creation of lift lines.

The structural buildup at rest and thixotropy of SCC can be evaluated by a rheometer, portable vane test, and inclined plane test [8] or by determining the variation in the results of workability tests (such as slump-flow and J-ring) of undisturbed samples with a rest time of over 1 h [5]. The consequences of these phenomena in multilayer casting of SCC involve not only unaesthetic lines and discolorations, but also deterioration of the mechanical performance. In general, it can be stated that the lower the viscosity and thixotropy of the mixture, the better the combination and bond strength between layers [6]. It should also be noted that the bond strength can be determined on a variety of specimens during different tests, and the magnitude of the thixotropy effect varies between them. 

Simple compression tests on specially prepared cylindrical core samples allow the shear strength of the interface between two concrete layers to be determined [7]. A reduction of the relative mechanical strength of more than 40% has been reported. Comparatively, two bond strength tests on eight SCC mixtures with different degrees of structural buildup at rest [8] showed that the bond strength determined using the direct shear stress is more adversely affected by multilayer casting than that evaluated using the slant shear strength. Flexural strength tests were also performed for mixtures of the same compositions [6]. In both studies, the tests were carried out for different delay times between successive layers of multilayer casting. The longer they were, the lower the bond strength was. For the delay time of 60 min, depending on the degree of structural buildup at rest, the residual bond strength was between 56% and 91%, 15% and 76%, and 56% and 80% for the slant shear test, direct shear test, and flexural strength test, respectively. The study also noted that the bond strength between successive layers can increase with an increase in the free drop of the top layer above freshly cast SCC due to the development of surface roughness and an interlock across the boundary.

In the case of the multilayer casting of SCC, the design guidelines [1,12] recommend that subsequent batches of SCC should be laid so that the previously poured mix can be plasticized on the surface, thus improving its proper combination with the newly laid mixture. This can be achieved by either surface mixing or by using low vibration. Other approaches involve increasing the mix pressure in the pipeline or increasing the height from which the mix is placed.

For most types of construction elements, the international guidelines [1,12,13] suggest using a single casting point for SCC mixes (preferably located at one of the edges) until the formwork is completely filled or reducing the number of casting points to the minimum and adjusting them to the rheological properties of the mix. The flow of the mixture at a certain distance is important as it enables proper self-venting and self-leveling. In the case of vertical elements (walls, columns, and deep beams), it is also possible to pump the mixture from the bottom of the formwork [14]. However, this technology requires a constant pump operation and even the shortest possible interruptions in concreting are not acceptable.

Given the fact that, in practice, it is rarely possible to carry out continuous concrete work and there is an increasingly widespread implementation of SCC, the impact of the delay time between placing successive layers of SCC on the bond strength in multilayer casting should be carefully verified, taking into account the recommended placement technologies. In this study, an experimental program was implemented to evaluate the effect of multilayer casting technology on the bond strength between two layers of SCC. The obtained results allowed us to present guidelines and suggestions for the multilayer casting of SCC, in order to reduce the impact of delays between successive layers on the mechanical strength and durability of the structure. These guidelines should be helpful for quality control and construction engineers when executing beam and wall elements with SCC.

## 2. Materials and Methods 

### 2.1. Concrete Mixture

One SCC mix, the composition of which is presented in Table 1, was adopted for experimental purposes. It was made with Portland ash cement (CEM II/B-V 32.5R) of strength class 32.5 N/mm^2^, a high early strength, and a density of 2.82 g/cm^3^. The mineral addition in the form of fly ash (FA) was of category A, which means that the loss on ignition was less than 5%, and it belonged to the category of fineness N. The declared density of the fly ash was 2.10 g/cm^3^. The aggregates met the requirements and were categorized according to the normative guidelines [15,16]. As for the coarse aggregate, two fractions of gravel were used, namely 2–8 mm and 8–16 mm. Both factions were of grading category G_C_85/20, according to [15]. The aggregate had a bulk density of 1.62 and 1.53 g/cm^3^, respectively, for 2–8 mm and 8–16 mm fractions. Natural sand of the fraction 0–2 mm was used as a fine aggregate. The sand was of grading category G_F_85, according to [15], and had a bulk density of 1.64 g/cm^3^. Polycarboxylic ether polymer superplasticizer was applied to ensure proper fluidity of the mixture. The density of the superplasticizer at a temperature of 20 °C was 1.07 g/cm^3^. 

The research assumed the production of SCC with an addition of fly ash. The mix proportions were design based on the literature [17] and previous experience of the authors. The total binder contribution was assumed to be 450 kg/m^3^, which is the minimum value recommended for SCC mixes. The ratio of fly ash content to the total amount of binder was assumed to be 20%. The water-to-binder ratio was set at 0.36. The total aggregate content was 1400 kg/m^3^, and the sand point ratio was maintained at 50%. The superplasticizer dosing was adjusted, in order to obtain proper fluidity and plastic viscosity of the SCC mix. 

### 2.2. Test Specimens

**Figure 1:**
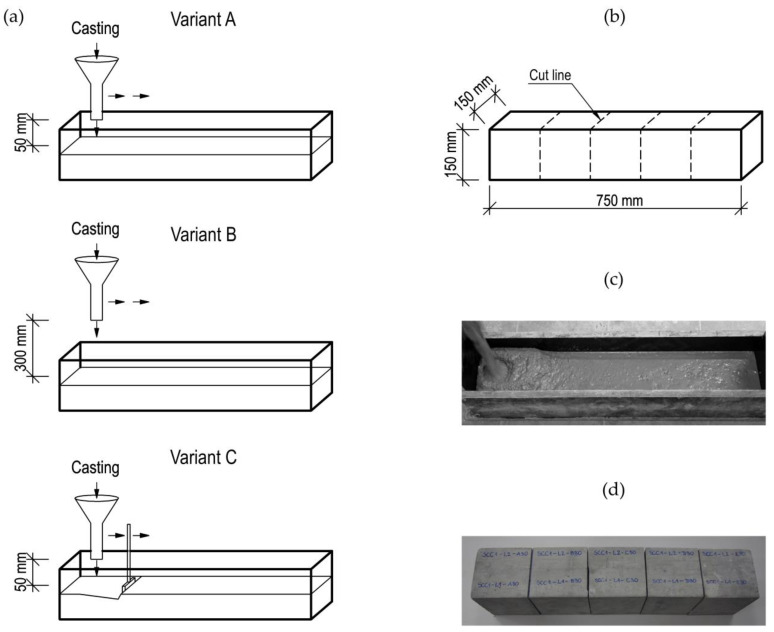
The test elements used in the studies: (**a**) A schematic view of multilayer casting variants; (**b**) a schematic view of a test element; (**c**) a variant of multilayer casting of an increased drop height; (**d**) a test element cut into modules.

Elements of dimensions 750 mm × 150 mm × 150 mm (length, height, and width) were molded for testing purposes. The beam element was designed so that it consisted of five basic modules in the form of a cube with dimensions of 150 mm × 150 mm × 150 mm (Figure 1b,d). Each specimen was made in two stages. In the first step, a layer of concrete from one batch (SCC-1) was placed up to half the height of the element, i.e., 75 mm. In the second phase, using the next concrete batch (SCC-2), the forms were filled up to the full height of 150 mm. Both batches were based on the same composition and produced using the same mixing process, which reflects the actual conditions of the multilayer casting of SCC generated by the mix supply gap to the construction site. The simulation of the interruption in the mixture casting was performed by postponing the time of the second part of the process. The delay times of the second layer of the mixture were 30, 45, and 60 min. For each period of time, different technologies of the preparation of the previously placed mix were used (Figure 1a). Variant A involved no interference with the first layer of the mixture. It was executed by placing the mixture from a container located about 5 cm above the surface of the previously laid layer. Variant B assumed restoration of a plastic state of the first layer of the mix as a result of placing the subsequent layer from the height of about 30 cm (Figure 1c). Variant C was based on the idea of a mechanical disturbance of the previously placed layer by surface mixing with a trowel and then laying the next batch of mix from the height of 5 cm. In each variant, the mixture was spread along the whole element, without setting one casting point.

**Figure 2:**
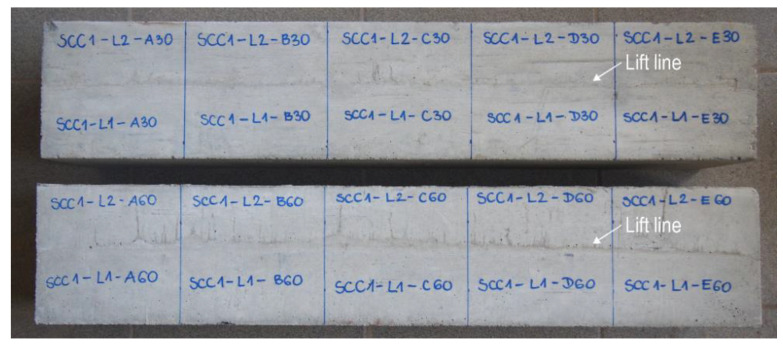
Test elements with visible lift lines (Variant A of placement technology).

The formwork was stripped after 3 days of concrete curing. The test elements were left in a stable position in laboratory conditions for the whole period of concrete ageing. Moreover, they were cared for through water sprinkling. On the surface of the elements made with the Variant A of placement technology, a lift line could be observed (Figure 2). The elements were cut into elementary parts after 21 days, and tests were carried out after 28 days. For each test series, two elements were made, which gave a total of 10 base specimens of a given model. Therefore, in total, 90 base modules were prepared. Additionally, 10 control samples made in one layer from each batch were prepared for compressive strength and splitting tensile strength testing.

### 2.3. Test Procedures

#### 2.3.1. Fresh Mix Property Tests

Fresh SCC mixes were tested in order to evaluate their flow properties. The rheological behavior of the fresh SCC was evaluated during three tests. A slump-flow test served as an investigation of both the flowability and plastic viscosity [18]. The measured parameters were the final slump-flow diameter and the time T_500_, which is related to a slump-flow time up to a diameter of 500 mm. The passing ability was tested using the L-box test [19]. This property was deduced from the L-box ratio. Finally, the Fresh Visual Segregation Index assessed after conducting the slump-flow test allowed us to determine the segregation resistance [20]. In the case of SCC-I, the rheological tests were performed immediately after the completion of mixing (t = 0 min). The SCC-II rheological tests were performed shortly before the application in the form, as well as 15 and 30 min after the completion of mixing (t = 0 min, t = 15 min, and t = 30 min, respectively). The batch was mixed during the whole period of time.

#### 2.3.2. Hardened Concrete Tests

During the concreting works, five reference samples were taken from each batch of concrete used in the test, in order to conduct a compressive strength test according to the procedure [21]. Moreover, five samples from each batch were prepared to determine the splitting tensile strength, in accordance with an appropriate procedure [22]. These accompanying tests were performed on cube specimens with dimensions of 150 mm × 150 mm × 150 mm, after 28 days of curing in laboratory conditions at a temperature of 20 °C and 95% relative humidity.

#### 2.3.3. Test of the Bond Strength in the Multilayer Casting of SCC

**Figure 3:**
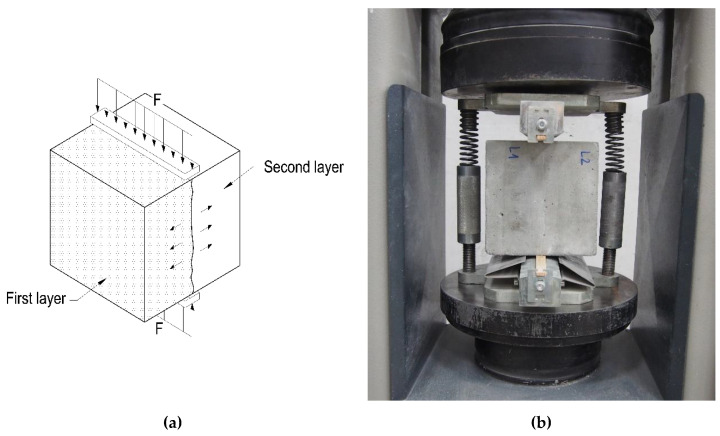
A view of (**a**) the test setup schematic and (**b**) a typical sample for bond strength tests on Standard Automatic compression tester, Controls, Compact-Line.

The bond strength between successive layers of SCC was examined on cubic samples with dimensions of 150 mm × 150 mm × 150 mm, which were subjected to splitting tension. The testing procedure was based on the guidelines given in the standard [22]. The tests were carried out after 28 days of curing in laboratory conditions. The interface between the two layers of the mixture was situated vertically, along the axis of applied forces. The scheme of the test setup is given in Figure 3. The bond strength of the joint was calculated as in the procedure used to determine the splitting tensile strength of concrete, according to the formula presented in Equation (1).

(1) $$f_{ct,sp}=\frac{2\cdot F}{\pi \cdot L\cdot d}$$

where *f_ct,sp_* is the splitting tensile strength, MPa;

*F* is the peak load, N;

*L* is the length of the sample contact line, mm;

and *d* is the declared cross-sectional dimension, mm.

## 3. Results

### 3.1. Fresh Mix Properties and Concrete Strength

The results of the flow properties of fresh SCC mixtures are presented in Table 2. The fresh property test results allowed us to classify both batches of the mixture as being of the same slump-flow class (SF2), and similar plastic viscosity (VS1 and VS2) and passing ability (PL2) classes. The slump-flow class SF2 lies in the range of 660–750 mm slump-flow diameters, and the limit between the viscosity classes VS1 and VS2 is T_500_ = 2 s. To qualify for the passing ability class PL2, mixtures are expected to have an L-box ratio of above 0.80 when tested in an L-box with three bars. The mixtures also had a similar stability, without any visible signs of segregation or bleeding, which is tantamount to obtaining the most favorable value of a fresh visual stability index, namely 0. 

Table 3 summarizes the results of compressive strength and splitting tensile strength tests of control samples cast in a single layer. The samples were made immediately after mixing and conducting fresh mix property tests. The results of batches did not differ significantly, which was a consequence of maintaining appropriate laboratory conditions during mixing and correct proportions of components. Small differences were the outcome of results scatter while sustaining one level of strength parameters.

### 3.2. Bond Strength Between Two Concrete Layers

Table 4 presents the mean results of splitting tensile strength tests carried out on the samples derived from the test elements. In addition to the average splitting tensile strength values, which were used as a criterion for the bond strength between successive layers, the standard deviation of the dataset is also shown. In general, the bond strength between the layers decreased with an extension of the delay time between successive layers. Only in the case of the samples made in Variant C with a 30 min delay time did the splitting tensile strength slightly increase. This phenomenon could have occurred due to the proper mixing of layers in Variant C of the multilayer casting technology and behavior similar to that of monolithic samples. Therefore, it may be possible to treat the technology as being similar to the monolithic one, and higher values of the results were associated with scatter.

The maximum reduction was obtained for Variant A in the whole scope of research. In the case of a 60 min delay time, the average bond strength reduction was 36%. In the case of Variant C, the results of the sample with a 60 min delay time were lower than those of the control sample by an average of less than 5%. For Variant B, an intermediate value of the decrease of nearly 13% was obtained. Table 4 also includes the reduction rate in bond strength with the delay time, which describes how rapidly the strength decreased in relation to one minute. Variant A, which involved no interference with the outer surface of the first layer, exhibited the most significant drop in time, with 0.0186 MPa/min. For the remaining two variants, a decrease of less than 0.01 MPa/min was recorded.

The most effective performance of Variant C corresponded to even and controlled interference across the entire contact surface through its mechanical disturbance. This resulted in an even improvement of the joint quality along the whole length of the element, rather than merely above the casting point. However, one should be aware that such interference may cause an unfavorable segregation phenomenon, and thus requires a controlled and planned procedure.

### 3.3. Effect of Multilayer Casting Variants on the Bond Strength 

**Figure 4:**
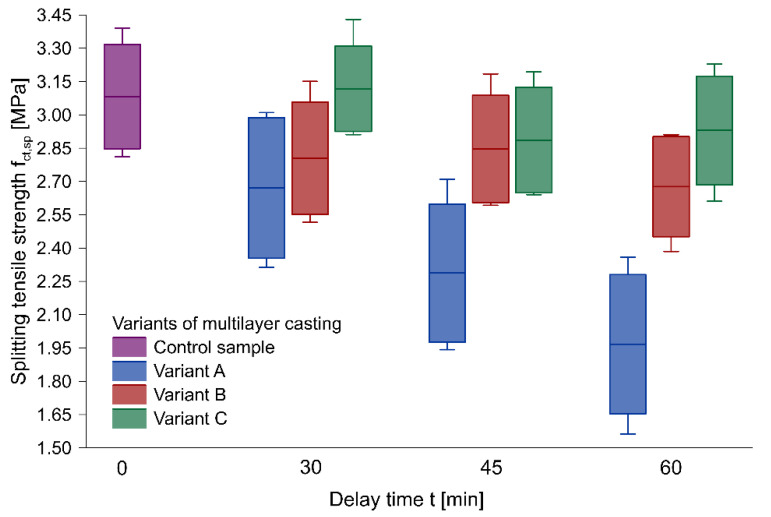
The relationship between the splitting tensile strength and delay time of successive layers.

Figure 4 illustrates the distribution of the results of the splitting tensile strength determined on the samples derived from the test elements, with regard to the delay time between layers and the placing technology, using box plots. The diagrams were based on an average value of strength parameters. The box was designed to represent a standard deviation of the results, and the whiskers defined the minimum and maximum values. It was noted that, for Variant A, the scatter of results was much greater compared to that of other methods, which, in the diagram, is represented by elongated boxes. Moreover, a significant decrease in the splitting tensile strength with delay time extension was observed in the case of this method when compared to the other variants. The increased scatter of the results recorded for Variant A compared to other multilayer casting technologies was caused, among other things, by the significantly lower splitting tensile strength of the outer samples in the beams of Variant A. Those samples acquired the smallest area of proper mixing of two layers. In the case of Variants B and C, the scatter of the results remained at a comparable level. A noteworthy example was the case of the element made in Variant C with a 30 min delay time between layers, for which no decrease in the splitting tensile strength was observed in comparison with the control sample. This phenomenon may be explained by the lack of a significant difference in the performance, as well as by behavior resembling a monolithic sample.

**Figure 5:**
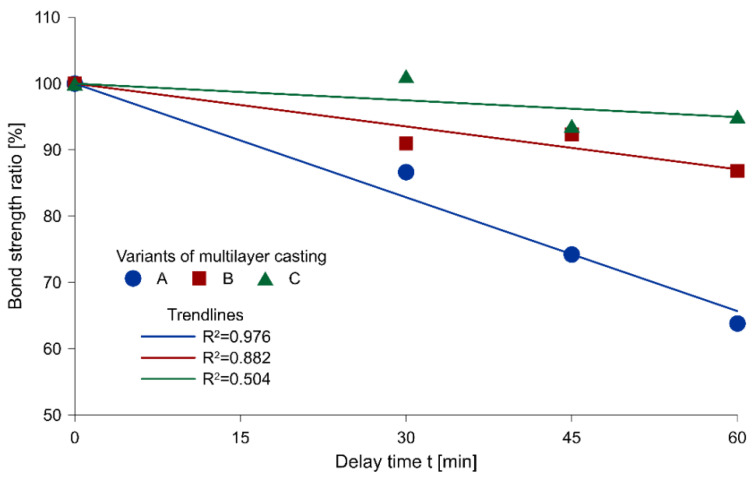
The relationship between the bond strength ratio and delay time of successive layers.

Figure 5 presents the relationship between the average bond strength ratio and the delay time for each variant of multilayer casting, with the corresponding linear trend lines. The bond strength ratio was adopted as a comparison of the mean of the results obtained from the samples of different delay times between layers to the values of control samples.

A decreasing tendency with an increasing delay time between successive layers of concrete, regardless of the multilayer casting technology, was observed throughout the entire scope of research. In the case of Variant A, the reduction was the largest, and the value of the coefficient of determination of R^2^ = 0.976 indicated a strong linear dependence. For the remaining two variants, the drop remained relatively minor and the distribution of results did not show a linear relationship (coefficient of determination R^2^ equal to 0.882 and 0.504 in Variants B and C, respectively).

A minimal reduction in strength was recorded in the case of Variant C, i.e., local plasticization of the mixture by mechanical disturbance of its upper layer. A slightly poorer performance was found in Variant B. However, dropping the mixture from a height did not disturb the surface of the first layer as effectively as it occurred due to mechanical interference with the surface.

### 3.4. Bond Failure Mechanism

**Figure 6:**
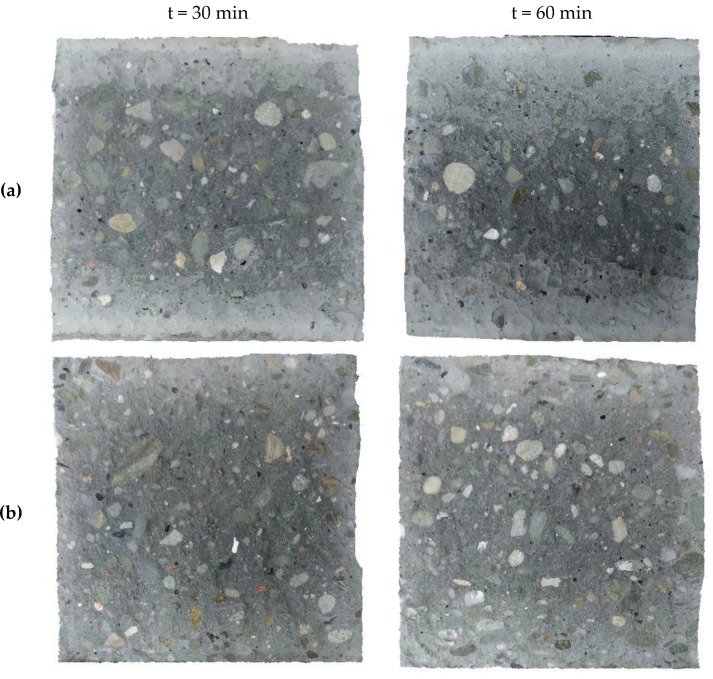

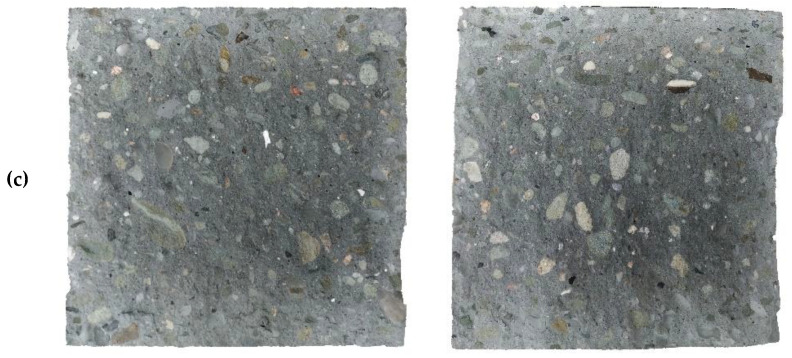
Examples of the surface of the first layer of specimens after splitting tensile strength tests: (**a**) Variant A; (**b**) Variant B; (**c**) Variant C.

An analysis of the condition of the surface after splitting tensile strength tests was carried out in order to explain the phenomena causing a reduction in the bond strength between two layers of SCC. Typical surface conditions of the first layer after the splitting tensile strength test of samples with a delay time of 30 and 60 min are shown in Figure 6.

The bond strength between the successive layers of the SCC mix is generated by the proper mixing of both layers and the level of structural buildup at rest of concrete mix used in the first layer. The mechanism of bond failure can be determined after strength tests with digital image processing [8,23] or using an optical measuring device [24]. To determine the failure mechanism of the successive layers of the SCC mixture and the effectiveness of their mixing for each placement technology, the surfaces of the samples were examined after splitting tensile strength tests, using their close-shot photos. The analysis was carried out using a Computer-Aided Design (CAD) program.

**Figure 7:**
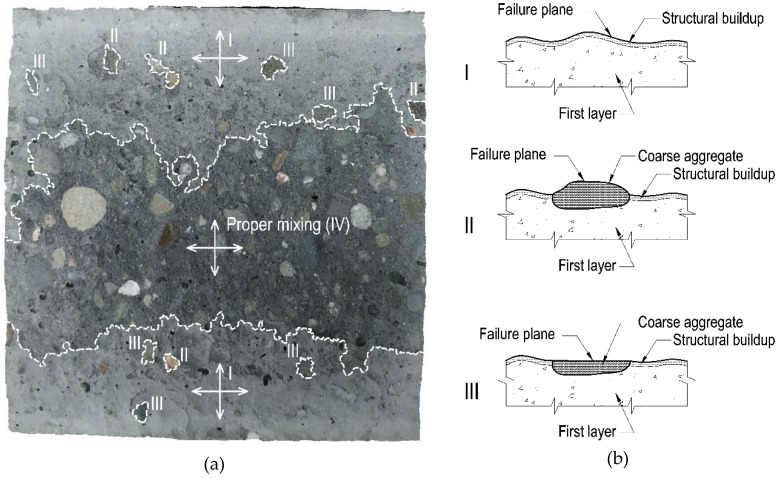
The surface of the first layer of a sample made with Variant A placement technology with a 60 min delay time between layers after a splitting tensile strength test: (**a**) Marked surfaces of various failure mechanisms; (**b**) description of the failure mechanism.

Figure 7 shows a representative surface of the sample (first layer, Variant A of placement technology, 60 min delay time) after failure with marked zones of different failure mechanisms. For this specimen, it is possible to clearly indicate all of the observed failure mechanisms in the research. There are four types of bond failure mechanism: I–destruction of the joint of two layers, together with the occurrence of structural buildup at rest of the first layer caused by its thixotropy; II–destruction through the first layer with the occurrence of structural buildup at rest and separation of the second layer from the coarse aggregate surface; III–destruction through the first layer with the occurrence of structural buildup at rest, with simultaneous exceeding of the tensile strength of the coarse aggregate; and IV–destruction through the surface of monolithic concrete (proper mixing of the layers).

In the case of Variant A of placement technology, the area of proper mixing of the layers was located in the middle section of the sample, where the fresh mixture had struck the partially stiffened first layer from a small height. The magnitude of this zone was dependent on the delay time. The longer the delay time between layers, the smaller the proper mixing zone was (Figure 6a). Within the other zones of the sample, the second layer spread freely after the surface of the first layer had stiffened. A sufficient shear stress did not occur to restore the plasticity of the first layer. Consequently, the second layer of the mixture did not mix with the first layer. Destruction in these zones was mainly a consequence of the separation of the contact between the layers passing through the stiffened surface or the coarse aggregates of the first layer. Therefore, the created joint was similar in its structure to a combination of a smooth surface hardened concrete and fresh concrete. An interfacial transition zone was formed at the point of contact between the batches of concrete, within which the failure surface of the composite element was found. The bond strength of this type of connection is low and, according to literature data [25], constitutes about 50% of the strength of the monolithic element. The research revealed that the larger the area of the intact first layer surface was, the smaller the bond strength between two layers was (Table 4, Figure 5).

In the case of Variant B and C of placement technology, proper mixing of the layers occurred, and the observed failure mechanism was mainly a type IV mechanism. However, at the edges of the samples, small zones of failure through the interfacial transition zone with the first layer partially stiffened—types I, II, and III of the failure mechanism—were observed (Figure 6b,c). Variants B and C of placement technology caused appropriate plasticization of the previously laid mixture, thus improving its mixing with the newly placed layer.

## 4. Discussion

This study provided a number of valuable findings and observations. The results enabled us to formulate recommendations for the multilayer casting of SCC, in order to reduce the impact of delays between successive layers on the mechanical strength and durability of the beam-type structure. These recommendations assumed plasticization of the mixture previously poured by superficial mechanical mixing (Variant C of placement technology). In the case of this variant, no significant loss of bond strength between successive layers was observed in the examined delay time range. The effective performance of Variant C was caused by even and controlled interference over the entire contact surface through its mechanical disturbance. It resulted in an improvement of the contact quality along the whole length of the element in a similar way, rather than only at the casting point of the mixture. In addition, this variant of placement technology helped to eliminate marks and discolorations on the concrete surface at the point of contact between layers (lift line). However, it should be remembered that such interference may cause an undesirable segregation phenomenon, and should thus be conducted in a controlled and planned manner.

The greatest decrease in bond strength between successive layers was obtained for Variant A of placement technology. For this variant, there was also a strictly linear relationship between bond strength reduction and the delay time between layers. The analysis of the condition of the failure surface indicated that in the variant, the mixing of subsequent layers was limited as a result of the formation of a stiffened surface on the first layer and the lack of a sufficient impact to restore its plastic state. 

It is not possible to quantitatively compare the results of this study with the literature research, since the effect of multilayer casting technology on the bond between successive layers is very dependent on the test method, sample shape, and type of contact surface. However, one can discuss comparable relationships, which were demonstrated in the studies [6,8]. In none of these tests was the surface mechanically disturbed. The tests were conducted for variants similar to Variant A and B of this paper. A reduction in the bond strength between layers of SCC, regardless of the mix composition, was observed when the delay time between batches was increased. Correspondingly, an improvement in the performance was noted when the height of the mixture drop was increased.

The presence of structural buildup at rest can be of significant relevance for the bond strength between successive layers when implementing multilayer casting in accordance with international guidelines [1,12,13]. The guidelines recommend placing concrete at a single casting point (preferably located at one of the edges) until the formwork is completely filled. The flow of the mixture at a certain distance is important, in order to allow for sufficient self-venting and self-leveling. This approach is well-suited for small components and/or a continuous mix supply. If there is an interruption in concrete works, the placement of subsequent layers of mix from a single casting point is inadvisable, especially for mixtures characterized by high structural buildup at rest. The mixture spreading over a large area after a stiffened first layer creates a lift line at the interface of the successive layers of the mixture. This leads to a reduction in the strength properties of the hardened concrete and its durability. Likewise, similar effects may occur in the case of Variant B of placement technology, where the mixture freely spreads across the partially solidified first layer, apart from the points of mixture discharge. Nevertheless, it is necessary to ensure efficient venting of the mixture by an adequate selection of rheological properties and concreting velocity.

Despite the interesting findings, the scope of the current studies is insufficient to draw definitive and extensive conclusions, and only preliminary ones can be suggested. Further research on the possibility of improving the bond strength between successive layers should be of interest and importance for construction engineers as it is a current problem on construction sites and the solutions given in the guidelines and recommendations differ in their results and may lead to a reduced strength layer in the element. The authors suggest that investigations should be extended to different self-compacting mixtures, different placing velocities, different layer thicknesses, other structural elements (slabs), and the appearance of reinforcement, in order to properly verify the effect of placement technology on the bond strength between successive layers. Moreover, one should note that the multilayer casting of concrete is a typical method of constructing massive structures, such as abutments or dams. One of the most promising and green approaches in such structures is the introduction of rock-filled concrete (RFC). The performance of RFC is significantly affected by the rheological properties of SCC, mainly the filling capacity [26,27]. The passing ability evaluated during an L-box test is sufficient to estimate the performance in typical reinforced concrete structures. However, when the obstacles are indefinable in shape (as in RFC), further studies should be addressed, considering the filling capacity during multilayer casting.

## 5. Conclusions

When constructing a concrete structure with breaks in concrete works, it is necessary to ensure that the subsequent layers are properly joined by mixing them together. In this study, three technological variants were tested to connect successive layers of SCC mix on beam elements (Variant A: free flow of the mix; Variant B: mixture drop from a height of 30 cm; Variant C: mechanical disturbance). The following conclusions and recommendations can be drawn on the basis of the experimental tests:

- In general, a reduction in bond strength between the layers was noted as the delay time between layers was extended. The most significant decrease was obtained in the case of Variant A in the entire range of research. For the delay times of 30, 45, and 60 min, the average reduction of bond strength in relation to the control sample for Variant A was 13%, 26%, and 36%, respectively, and for Variant B, was 8.8%, 7.5%, and 13%, respectively. In the case of Variant C, hardly any change was obtained for the 30 min delay time and for the 45 and 60 min delay times, the decrease was 6% and 5%, respectively;
- The analysis of the failure surface condition showed that in Variant A, the proper mixing of subsequent layers is limited. This resulted from the structural buildup at rest of the first layer and the lack of a sufficient impact required for its plasticization. The larger the area of the intact surface of the first layer, the lower the bond strength between two layers. Variants B and C of placement technology caused the previously laid mixture to properly restore a plastic state on the surface, thus enabling its efficient mixing with the new one. In these technologies, no visible discolorations and traces were observed at the contact of layers—the so-called lift line;
- Based on the investigations, it is recommended that subsequent layers of SCC be placed so that the previously laid mix can be superficially plasticized, thus facilitating its bonding to the new layer. It is suggested that the method of either mechanical surface mixing or increasing the height from which the next batch of SCC is poured should be applied. If the variant of an increased casting height is adopted, the mix should be dropped over the entire surface of the component without a fixed single casting point.

## Figures and Tables

**Table 1:** Composition of the mixture.

Constituent Materials	Composition [kg/m^3^]
Cement CEM II/B-V 32.5R	360
Water	160
Sand 0–2 mm	700
Gravel aggregate 2–8 mm	350
Gravel aggregate 8–16 mm	350
Fly ash	90
Superplasticizer	2.8

**Table 2:** Fresh property test results.

Mix Batch	Delay Time	Slump-Flow (mm)	Slump-Flow Class	Slump-Flow Time T_500_ (s)	Viscosity Class	Fresh Visual Stability Index	L-Box Ratio	L-Box Class
SCC-I	t = 0 min	685	SF2	1.7	VS1	0	0.90	PL2
SCC-II	t = 0 min	710	SF2	1.9	VS1	0	0.92	PL2
SCC-II	t = 15 min	695	SF2	2.1	VS2	0	0.89	PL2
SCC-II	t = 30 min	680	SF2	2.5	VS2	0	0.87	PL2

**Table 3:** Results of compressive strength and splitting tensile strength tests.

Mix Batch	Compressive Strength		Splitting Tensile Strength	
	f_cc_ (MPa)	SD ^1^ (MPa)	f_ct,sp_ (MPa)	SD (MPa)
SCC-I	56.5	1.808	2.97	0.214
SCC-II	53.3	2.292	3.18	0.204

^1^ Standard deviation.

**Table 4:** Splitting tensile strength test results of multilayer cast samples.

Multilayer Casting Variant	Delay Time between Successive Layers								Reduction Rate in Bond Strength (MPa/min)
	t = 0 min ^1^		t = 30 min		t = 45 min		t = 60 min		
	f_ct,sp_ (MPa)	SD ^2^ (MPa)	f_ct,sp_ (MPa)	SD (MPa)	f_ct,sp_ (MPa)	SD (MPa)	f_ct,sp_ (MPa)	SD (MPa)	
A	3.08	0.210	2.67	0.284	2.29	0.278	1.97	0.280	0.0186
B			2.81	0.226	2.85	0.216	2.68	0.201	0.0068
C			3.12	0.172	2.89	0.213	2.93	0.218	0.0025

^1^ The mean value of the splitting tensile strength for samples made of both self-compacting concrete (SCC) mix batches. ^2^ Standard deviation.
